# Impact of vericiguat on baroreflex-mediated sympathetic circulatory regulation: An open-loop analysis

**DOI:** 10.1371/journal.pone.0286767

**Published:** 2023-08-11

**Authors:** Aimi Yokoi, Toru Kawada, Shohei Yokota, Midori Kakuuchi, Hiroki Matsushita, Akitsugu Nishiura, Meihua Li, Kazunori Uemura, Joe Alexander, Ryou Tanaka, Keita Saku

**Affiliations:** 1 Department of Cardiovascular Dynamics, National Cerebral and Cardiovascular Center, Suita, Osaka, Japan; 2 Tokyo University of Agriculture and Technology, Fuchu, Tokyo, Japan; 3 Medical and Health Informatics, NTT Research, Inc., Sunnyvale, CA, United States of America; Kyoto University Graduate School of Medicine Faculty of Medicine: Kyoto Daigaku Daigakuin Igaku Kenkyuka Igakubu, JAPAN

## Abstract

**Aims:**

To quantify *in vivo* the effects of the soluble guanylate cyclase (sGC) stimulator, vericiguat, on autonomic cardiovascular regulation in comparison with the nitric oxide (NO) donor, sodium nitroprusside.

**Methods:**

In anesthetized Wistar–Kyoto rats, baroreflex-mediated changes in sympathetic nerve activity (SNA), arterial pressure (AP), central venous pressure (CVP), and aortic flow (AoF) were examined before and during the intravenous continuous administration (10 μg·kg^−1^·min^−1^) of vericiguat or sodium nitroprusside (n = 8 each). Systemic vascular resistance (SVR) was calculated as SVR = (AP–CVP) / AoF.

**Results:**

Neither vericiguat nor sodium nitroprusside affected fitted parameters of the baroreflex-mediated SNA response. Both vericiguat and sodium nitroprusside decreased the AP mainly through their peripheral effects. Vericiguat halved the slope of the SNA–SVR relationship from 0.012 ± 0.002 to 0.006 ± 0.002 mmHg·min·mL^−1^·%^−1^ (P = 0.008), whereas sodium nitroprusside caused a near parallel downward shift in the SNA–SVR relationship with a reduction of the SVR intercept from 1.235 ± 0.187 to 0.851 ± 0.123 mmHg·min/mL (P = 0.008).

**Conclusion:**

Neither vericiguat nor sodium nitroprusside significantly affected the baroreflex-mediated SNA response. The vasodilative effect of vericiguat became greater toward high levels of SNA and AP, possibly reflecting the increased sGC sensitivity to endogenous NO. By contrast, the effect of sodium nitroprusside was more uniform over the range of SNA. These results help better understand cardiovascular effects of vericiguat.

## Introduction

Nitric oxide (NO) produced by vascular endotherial cells reaches vascular smooth muscles and stimulates soluble guanylate cyclase (sGC), which increases cyclic guanosine monophosphate (cGMP) to dilate the vasculature. In cardiovascular diseases such as heart failure, reactive oxygen species (ROS) reduce NO production and promote myocardial and vascular damage resulting in further deterioration of the disease condition [1]. Although NO donors supply NO to stimulate the NO-sGC-cGMP pathway, organic nitrates such as nitroglycerin can induce NO tolerance [2, 3]. Sodium nitroprusside (SNP) spontaneously liberates NO but can also induce NO tolerance [4, 5]. The short half-life of SNP makes it appropriate for use as an acute therapy but hinders its chronic application. sGC stimulators and activators are a new class of drugs that directly act on sGC, an emerging therapeutic target for cardiopulmonary disease [6]. The sGC stimulators bind to NO-sensitive heme-containing sGC, whereas the sGC activators bind to NO-insensitive heme-free sGC. The sGC stimulators can act independently of NO and also in synergy with NO [7, 8]. Several clinical trials have shown benefits of vericiguat, a sGC stimulator, in patients with heart failure [9–11].

The treatment of heart failure has improved and the standard of care has updated, but the prognosis of heart failure remains unfavorable. There is a requirement for better heart failure treatment. It has been reported that the major event preceding the deterioration of recurrent heart failure is a significant increase in systemic vascular resistance (SVR) [12]. Decreasing SVR in heart failure lowers cardiac afterload, leading to increased cardiac output and peripheral perfusion [13, 14]. Mandry et al. reported that increased ejection fraction was associated with decreased SVR [14]. According to Follmann et al., vericiguat blocks contraction of isolated arteries in normal and nitrate-tolerant rabbits [7]. Vericiguat may alleviate the increase of SVR *in vivo* as a vasodilator.

Although vasodilation is expected from its key mechanism of action, whether or not vericiguat significantly modifies autonomic cardiovascular regulation *in vivo* remains to be quantitatively analyzed. The sympathetic limb of the autonomic nervous system plays a critical role in controlling SVR. Under normal physiological conditions, a decrease in arterial pressure (AP) increases sympathetic nerve activity (SNA) via the arterial baroreflex. Hence, the vasodilative effect of a given drug is partly counterbalanced by the baroreflex-mediated, sympathetic vasoconstriction. To circumvent such a confounding negative feedback effect, we have applied a framework of an open-loop analysis to the carotid sinus baroreflex system [15]. In this framework, the carotid sinus baroreflex system is divided into two principal subsystems: the neural arc subsystem, which represents the input–output relationship between baroreceptor pressure inputs and SNA, and the peripheral arc subsystem, which represents the relationship between SNA and AP [16]. The aim of the present study is twofold: 1) to quantify the vasodilative effect of vericiguat at different levels of SNA, and 2) to elucidate the effect of vericiguat on baroreflex-mediated regulation of SNA. Moreover, the effects of vericiguat on sympathetic cardiovascular regulation were compared with those of SNP.

## Materials and methods

This study conforms to the "Guiding Principles for the Care and Use of Animals in Physiological Sciences" approved by the Japanese Physiological Society. All experimental protocols were reviewed and approved by the Animal Subjects Committee of the National Cardiovascular Center (No. 21009, 22033). Male Wistar–Kyoto rats were purchased from Japan SLC.

### Preparation

Each rat was anesthetized by intraperitoneal injection (2 mL/kg) of a mixed anesthetic of urethane (250 mg/mL) and α-chloralose (40 mg/mL) and artificially ventilated with oxygenated air. A venous catheter was inserted into the left femoral vein and advanced to the inferior vena cava to measure central venous pressure (CVP). Another venous line was inserted from the right femoral vein for drug administration. A catheter-tip micromanometer (SPR-320, Millar, USA) was introduced from the right common carotid artery to measure AP. Heart rate (HR) was detected from a body surface electrocardiogram (AT-601G, Nihon Kohden, Japan). The anesthetic mixture was diluted 18-fold with physiological saline and administered continuously (2–3 mL·kg^−1^·h^−1^) to maintain anesthesia. Ringer’s lactate solution was infused (4 mL·kg^−1^·h^−1^) throughout the experiment to maintain fluid balance. The body temperature of the rats was maintained between 37°C and 38°C using a warming mat and a lamp.

Through a left flank incision, a pair of stainless-steel wire electrodes (AS633, Cooner Wire, USA) were attached to the postganglionic branch of the splanchnic sympathetic nerve. The nerve and electrodes were fixed and insulated with silicone adhesive (Kwik-Sil, World Precision Instruments, USA). The electrical signal was amplified using a biological amplifier and band-pass filtered between 150 and 1,000 Hz (AB-610J, Nihon Kohden, Japan). The signal was then full-wave rectified and low-pass filtered with a cut-off frequency of 30 Hz to quantify SNA. At the end of the experiment, a ganglionic blocker, hexamethonium bromide (FUJIFILM Wako Pure Chemical, Japan), was injected intravenously (60 mg/kg) to confirm SNA disappearance and determine the noise level.

The carotid sinus baroreceptor areas were isolated from the systemic circulation [17, 18], and carotid sinus pressure (CSP) was controlled using a servo-pump system (ET-126, Labworks, USA). To minimize reflex effects from the cardiopulmonary region and the aortic arch, the vagal nerves and the depressor nerves were sectioned in the neck region. Following the baroreceptor isolation, a flow probe (MA-2PSB, Transonic Systems, USA) was placed around the ascending aorta through a midline thoracotomy to measure aortic flow (AoF) (TS-420, Transonic Systems, USA).

### Protocol

CSP was first decreased to 60 mmHg for 5 minutes, then increased stepwise in increments of 20 mmHg every minute to 180 mmHg. The 11-min CSP input sequence was repeated and designated as S1 through S4. From 1 minute after the completion of S2, an intravenous continuous administration of vericiguat or SNP was started.

Vericiguat protocol (n = 8 rats, 345–380 g): Vericiguat (BAY 1021189, MedChemExpress, USA) was dissolved with dimethyl sulfoxide (DMSO, FUJIFILM Wako Pure Chemical Corporation, Japan) at 1 mg/100 μL, and diluted with 1:1 volume of polyethylene glycol 200 and physiological saline to obtain the solution of 600 μg·kg^−1^·mL^−1^. The infusion rate was 1 mL/h, achieving the dose of 600 μg·kg^−1^·h^−1^ (10 μg·kg^−1^·min^−1^ or 23.5 nmol·kg^−1^·min^−1^). Because there was little available information on the use of vericiguat for continuous intravenous administration in rats, the dose used was adopted from the high dose of BAY 41–2272 (an agonist for sGC) used in anesthetized dogs [19]. The infusion volume was counterbalanced by reducing the infusion rate of the Ringer’s lactate solution by 1 mL/h.

SNP protocol (n = 8 rats, 326–385 g): SNP (Maruishi Pharmaceutical, Japan) was purchased as a solution of 3 mg/mL, and a regime comparable to vericiguat was adopted; i.e., 100 μL of DMSO was added per 1 mg of SNP, and diluted with 1:1 volume of polyethylene glycol 200 and physiological saline to the concentration of 600 μg·kg^−1^·mL^−1^ (10 μg·kg^−1^·min^−1^ or 33.6 nmol·kg^−1^·min^−1^). This solution was infused at 1 mL/h while the infusion rate of Ringer’s lactate solution was reduced by 1 mL/h.

### Data analysis

Data were digitized with a 16-bit analog-to-digital converter (AIO AD16-16(PCI)EV, Contec, Japan) and recorded at 1,000 Hz on a laboratory computer system using custom-made software. The SNA, AP, HR, AoF, and CVP signals for the last 10 s of each CSP step (from 49 to 59 s to be exact) were averaged to obtain steady-state response values at each CSP level. Because the absolute amplitude of SNA varied depending on recording conditions across animals, the SNA was standardized in each animal based on values at CSP of 60 mmHg during S2 (100%) and after ganglionic blockade (0%). SVR was calculated from SVR = (AP–CVP) / AoF.

**Fig 1:**
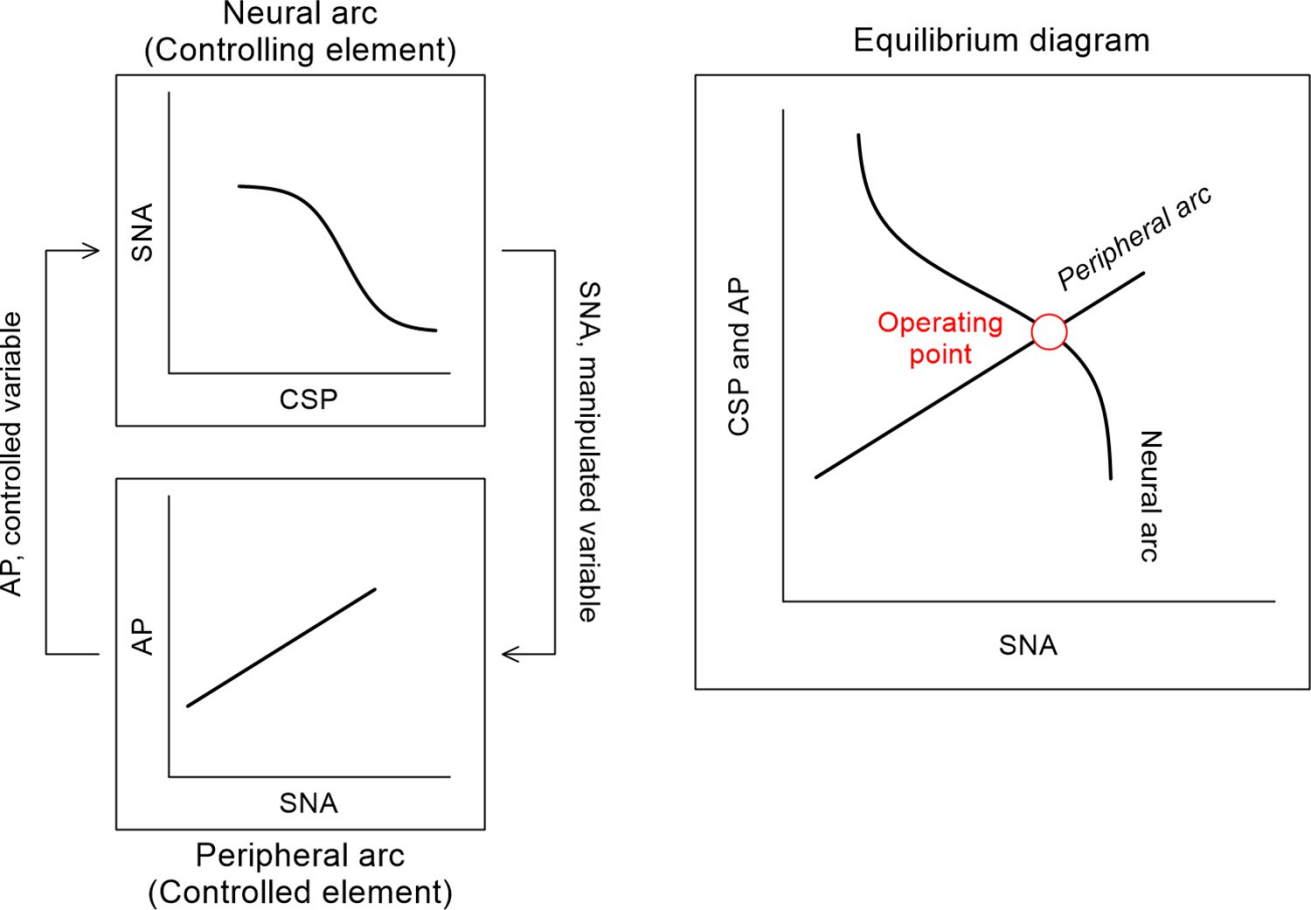
A conceptual figure representing the baroreflex equilibrium diagram analysis. The baroreflex equilibrium diagram is obtained by plotting the neural arc (CSP–SNA relationship) and peripheral arc (SNA–AP relationship) on the plane of pressure versus SNA. The operating point of the carotid sinus baroreflex is determined from the intersection of the neural and peripheral arcs on the baroreflex equilibrium diagram.

The AP, HR, and SNA responses as a function of CSP were quantified using the following 4-parameter logistic function [20].

$$y=\frac{P_{1}}{1+exp[P_{2}(CSP-P_{3})]}+P_{4}$$ (1)

where y is the output variable; P_1_, P_2_, P_3_, and P_4_ are the response range, slope coefficient, midpoint pressure on the CSP axis, and lower limit of the sigmoid curve, respectively. The AP, SVR, and AoF as a function of SNA were quantified using linear regression.

y=Intercept+Slope×SNA (2)

where y is the output variable. The baroreflex equilibrium diagram was obtained by plotting the neural arc (CSP–SNA relationship) and peripheral arc (SNA–AP relationship) on the plane of pressure versus SNA (Fig 1) [21–23]. The operating point of the carotid sinus baroreflex was determined from the intersection of the neural and peripheral arcs on the baroreflex equilibrium diagram.

### Statistical analysis

Data are presented as mean ± SE values. In each drug protocol, the data during S2 served as baseline, and the drug effect was evaluated using the data during S4. Parameters were compared between S2 and S4 using the Wilcoxon signed-rank test [24]. The differences were considered statistically significant when P < 0.05.

## Results

**Fig 2:**
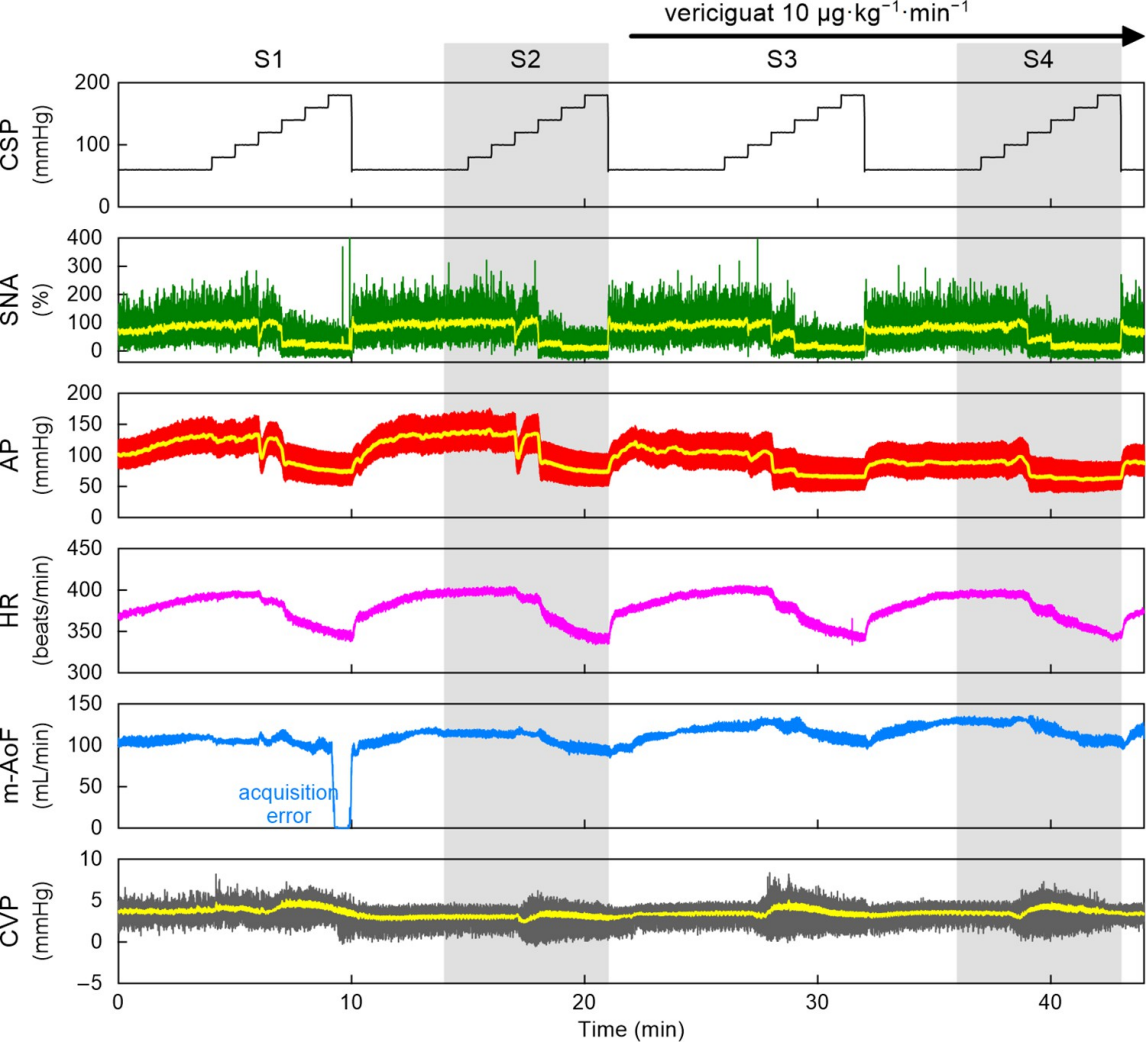
An example time series obtained from one rat in the vericiguat protocol. CSP was changed in a stepwise manner with a step duration of 60 s. The input sequence was repeated and designated as S1 through S4. An increase in CSP decreased SNA, AP, HR, and m-AoF. CVP did not change significantly with the increase in CSP. From 1 min after the completion of S2, an intravenous administration of vericiguat was started and continued throughout the end of the protocol. The effect of vericiguat was evaluated in S4. Vericiguat decreased AP without significant changes in the SNA and HR responses. The CSP and HR are presented as 10-Hz resampled signals. The green and yellow lines in the SNA plot indicate 10-Hz resampled and 2-s moving averaged signals, respectively. The red and yellow lines in the AP plot indicate 100-Hz resampled and 2-s moving averaged signals, respectively. The m-AoF is presented as 2-s moving averaged signals. CSP, carotid sinus pressure; SNA, sympathetic nerve activity; AP, arterial pressure; HR, heart rate; m-AoF, mean aortic flow; CVP, central venous pressure.

Fig 2 represents typical time series obtained from the vericiguat protocol. Under the baseline conditions (S2), an increase in CSP decreased SNA, AP, HR, and mean AoF (m-AoF). CVP did not change significantly with the increase in CSP, and the CVP response was not consistent among animals. From 1 min after the completion of S2, the intravenous continuous administration of vericiguat was started. The highest AP during low levels of CSP was decreased in S4 compared with that in S2. The SNA and HR responses were not much different between S2 and S4. The m-AoF slightly increased in S4 compared with that in S2.

**Fig 3:**
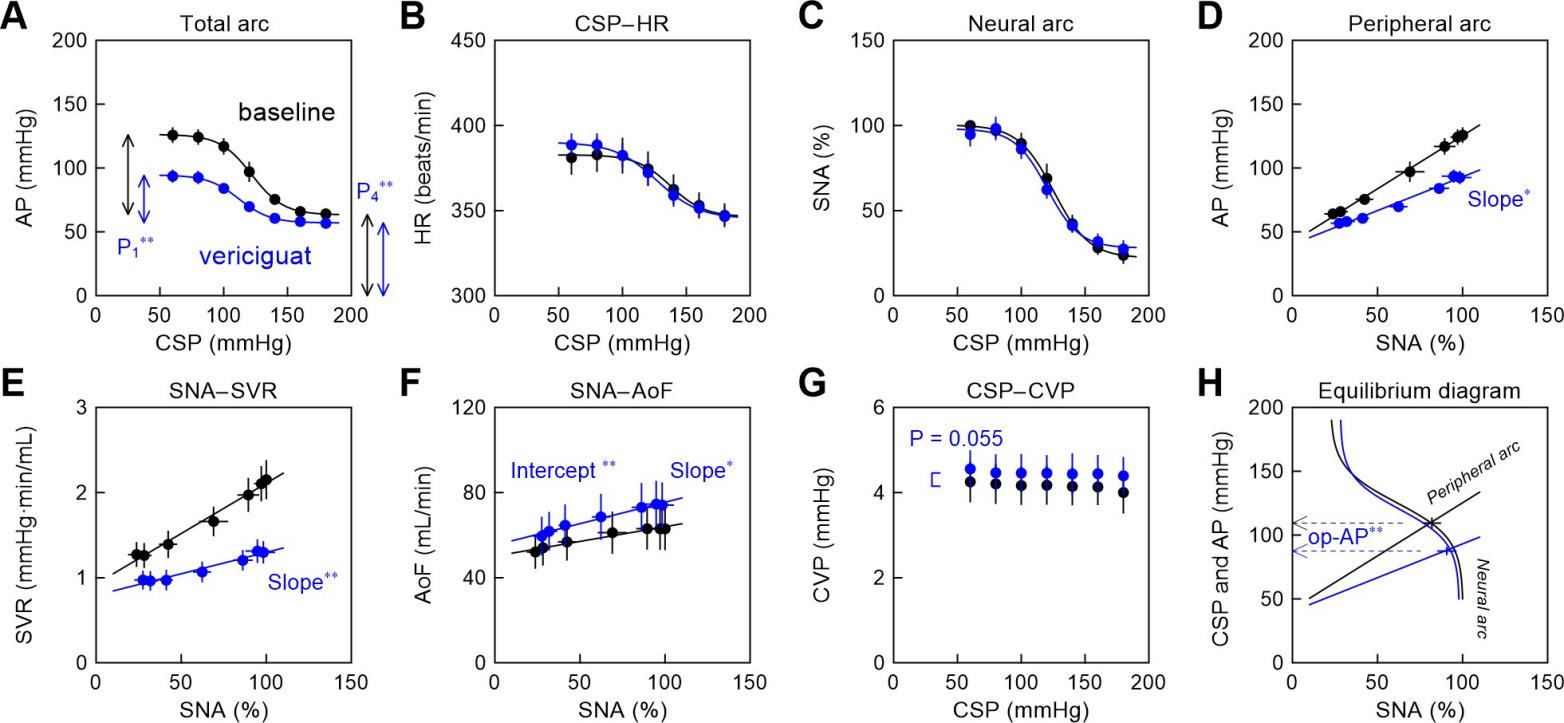
Hemodynamic changes and baroreflex equilibrium diagram during administration of vericiguat. Group-averaged relationships of the total arc (A), CSP–HR relationship (B), neural arc (C), peripheral arc (D), SNA–SVR relationship (E), SNA–AoF relationship (F), CSP–CVP relationship (G), and the baroreflex equilibrium diagram constructed from the mean curve of the neural arc and the mean line of the peripheral arc (H) in the vericiguat protocol. In panels A–G, the data points are presented as mean ± SE values. In panel H, the operating point is displayed as mean ± SE values. P_1_ and P_4_ indicate the response range and the lower limit, respectively. The symbol * and ** indicate statistical significances at P < 0.05 and P < 0.01, respectively, by the Wilcoxon signed-rank test. In panel G, the P value was derived from the Wilcoxon signed-rank test. CSP, carotid sinus pressure; AP, arterial pressure; HR, heart rate; SNA, sympathetic nerve activity; SVR, systemic vascular resistance; AoF, aortic flow; CVP, central venous pressure; op-AP, AP at the operating point.

Fig 3 and Table 1 summarize the effects of vericiguat on the baroreflex-mediated responses. Vericiguat significantly narrowed the range of the AP response (P_1_) and decreased the lower limit of the sigmoid curve (P_4_) in the total arc (Fig 3A), whereas it did not significantly affect the CSP–HR relationship (Fig 3B) or the neural arc (Fig 3C). Vericiguat significantly decreased the slope of the peripheral arc (Fig 3D) with a reduction in the slope of the SNA–SVR relationship (Fig 3E) and slight increases in the slope and intercept of the SNA–AoF relationship (Fig 3F). Vericiguat marginally increased the averaged CVP (Fig 3G). On the baroreflex equilibrium diagram (Fig 3H), the intersection between the neural and peripheral arcs gives the operating point. Vericiguat significantly decreased the AP at the operating point due to the reduction in the slope of the peripheral arc.

**Table 1:** Effects of vericiguat on fitted parameters relating to the baroreflex-mediated sympathetic cardiovascular regulation.

	Baseline	Vericiguat	P-value
Total arc			
P_1_, mmHg	63.3 ± 4.8	37.6 ± 4.1	0.008
P_2_, mmHg^−1^	0.096 ± 0.019	0.105 ± 0.023	0.461
P_3_, mmHg	119.7 ± 3.0	113.4 ± 2.7	0.055
P_4_, mmHg	64.0 ± 3.2	56.9 ± 2.2	0.008
HR control			
P_1_, beats/min	36.5 ± 4.7	43.9 ± 4.6	0.078
P_2_, mmHg^−1^	0.092 ± 0.009	0.075 ± 0.011	0.313
P_3_, mmHg	129.5 ± 7.9	125.6 ± 3.3	0.195
P_4_, beats/min	347.2 ± 6.4	346.0 ± 5.0	0.945
Neural arc			
P_1_, %	78.6 ± 5.2	70.5 ± 8.0	0.313
P_2_, mmHg^−1^	0.110 ± 0.024	0.104 ± 0.016	0.945
P_3_, mmHg	121.7 ± 5.8	120.9 ± 4.1	0.547
P_4_, %	23.8 ± 5.1	27.7 ± 4.8	0.383
Peripheral arc			
Intercept, mmHg	42.1 ± 7.6	37.6 ± 6.9	0.313
Slope, mmHg/%	0.820 ± 0.078	0.562 ± 0.084	0.016
SNA–SVR			
Intercept, mmHg·min/mL	0.875 ± 0.191	0.732 ± 0.147	0.195
Slope, mmHg·min·mL^−1^·%^−1^	0.012 ± 0.002	0.006 ± 0.002	0.008
SNA–AoF			
Intercept, mL/min	51.2 ± 7.6	56.0 ± 8.7	0.008
Slope, mL·min^−1^·%^−1^	0.121 ± 0.029	0.192 ± 0.019	0.039
Operating point			
AP, mmHg	109.4 ± 3.9	87.6 ± 2.8	0.008
SNA, %	81.9 ± 5.1	90.7 ± 5.3	0.461

Data are means ± SE (n = 8 rats). P_1_, response range; P_2_, slope coefficient; P_3_, midpoint pressure; P_4_, lower limit; SNA, sympathetic nerve activity; SVR, systemic vascular resistance; AoF, aortic flow; AP, arterial pressure. The P-values were derived from the Wilcoxon signed-rank test.

**Fig 4:**
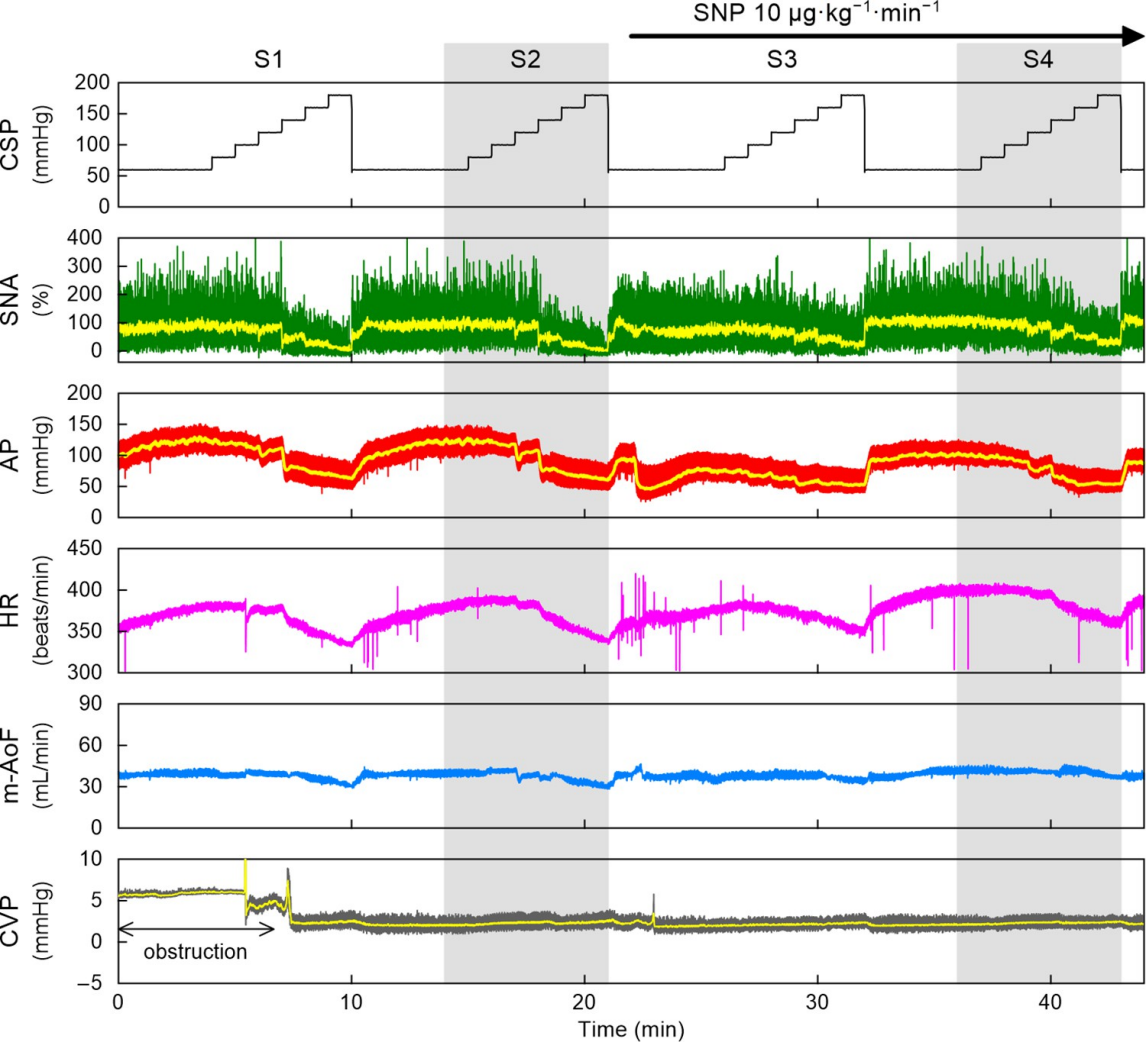
An example time series obtained from one rat in the SNP protocol. CSP was changed in a stepwise manner with a step duration of 60 s. The input sequence was repeated and designated as S1 through S4. An increase in CSP decreased SNA, AP, HR, and m-AoF. CVP did not change significantly with the increase in CSP. From 1 min after the completion of S2, an intravenous administration of SNP was started and continued throughout the end of the protocol. The effect of SNP was evaluated in S4. SNP decreased AP without significant changes in the SNA and HR responses. The CSP and HR are presented as 10-Hz resampled signals. The green and yellow lines in the SNA plot indicate 10-Hz resampled and 2-s moving averaged signals, respectively. The red and yellow lines in the AP plot indicate 100-Hz resampled and 2-s moving averaged signals, respectively. The m-AoF is presented as 2-s moving averaged signals. CSP, carotid sinus pressure; SNA, sympathetic nerve activity; AP, arterial pressure; HR, heart rate; m-AoF, mean aortic flow; CVP, central venous pressure; SNP, sodium nitroprusside.

Fig 4 represents typical time series obtained from the SNP protocol. Under the baseline conditions (S2), an increase in CSP decreased SNA, AP, HR, and m-AoF. CVP did not change significantly with the increase in CSP. From 1 min after the completion of S2, the intravenous continuous administration of SNP was started. The AP dropped acutely at the onset of the SNP administration. The highest AP during low levels of CSP was decreased in S4 compared with that in S2. The HR was slightly higher during S4 than during S2 in this rat, but this observation was not consistent across the group. SNP did not significantly affect the SNA or m-AoF response.

**Fig 5:**
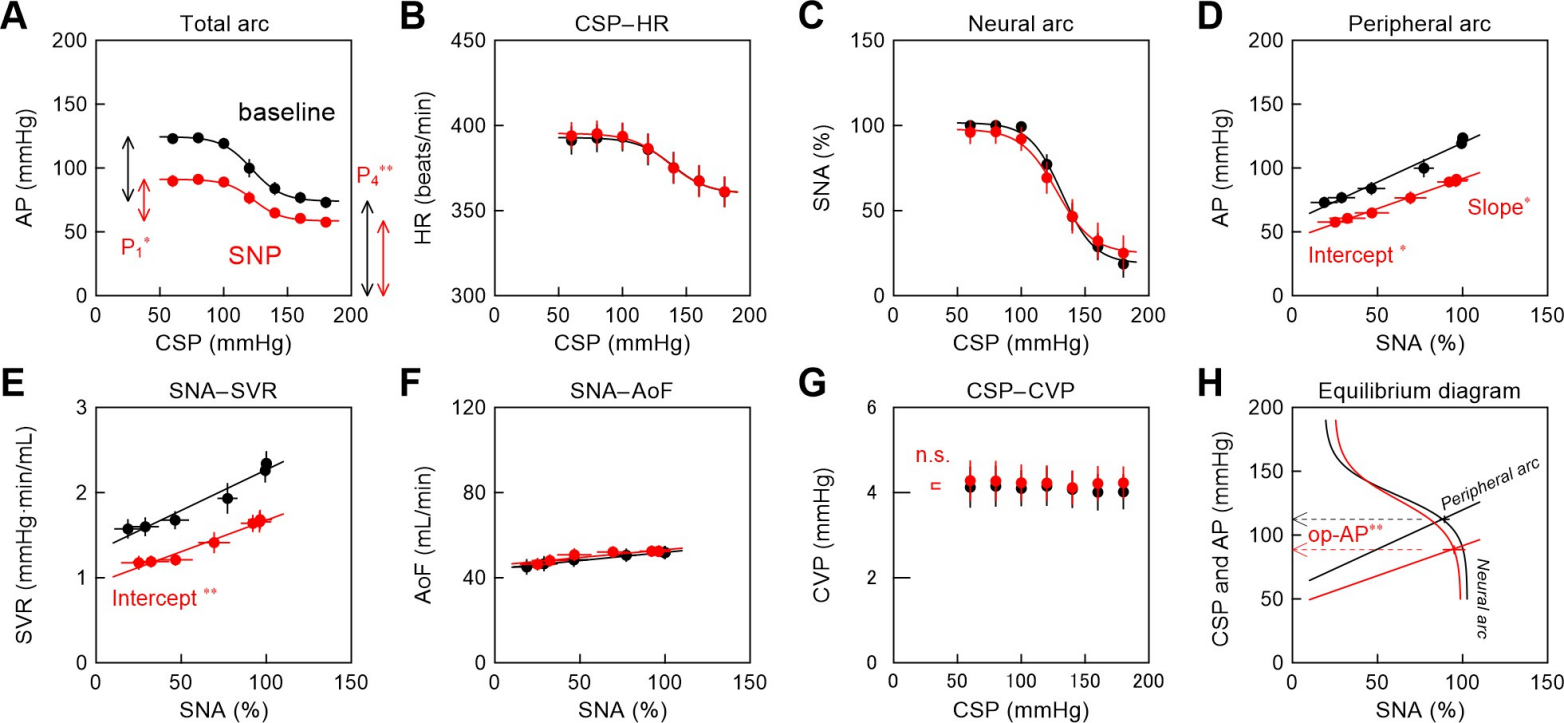
Hemodynamic changes and baroreflex equilibrium diagram during administration of SNP. Group-averaged relationships of the total arc (A), CSP–HR relationship (B), neural arc (C), peripheral arc (D), SNA–SVR relationship (E), SNA–AoF relationship (F), CSP–CVP relationship (G), and the baroreflex equilibrium diagram constructed from the mean curve of the neural arc and the mean line of the peripheral arc (H) in the SNP protocol. In panels A–G, the data points are presented as mean ± SE values. In panel H, the operating point is displayed as mean ± SE values. P_1_ and P_4_ indicate the response range and the lower limit, respectively. The symbol * and ** indicate statistical significances at P < 0.05 and P < 0.01, respectively, by the Wilcoxon signed-rank test. n.s., not significant by the Wilcoxon signed-rank test; CSP, carotid sinus pressure; AP, arterial pressure; HR, heart rate; SNA, sympathetic nerve activity; SVR, systemic vascular resistance; AoF, aortic flow; CVP, central venous pressure; op-AP, AP at the operating point; SNP, sodium nitroprusside.

Fig 5 and Table 2 summarize the effects of SNP on the baroreflex-mediated responses. SNP significantly decreased P_1_ and P_4_ in the total arc (Fig 5A), whereas it did not significantly affect the CSP–HR relationship (Fig 5B) or the neural arc (Fig 5C). SNP caused a downward shift in the peripheral arc with reductions in the slope and intercept (Fig 5D). During the SNP administration, the SNA–SVR relationship showed a near parallel downward shift (Fig 5E), whereas the SNA–AoF relationship hardly changed (Fig 5F). SNP did not significantly affect the averaged CVP (Fig 5G). The baroreflex equilibrium diagram indicates that SNP significantly decreased the AP at the operating point due to the change in the peripheral arc (Fig 5H).

**Table 2:** Effects of sodium nitroprusside (SNP) on fitted parameters relating to the baroreflex-mediated sympathetic cardiovascular regulation.

	Baseline	SNP	P-value
Total arc			
P_1_, mmHg	50.4 ± 2.9	32.9 ± 3.7	0.016
P_2_, mmHg^−1^	0.112 ± 0.023	0.153 ± 0.033	0.547
P_3_, mmHg	123.6 ± 3.3	120.3 ± 4.0	0.641
P_4_, mmHg	73.8 ± 3.7	58.8 ± 2.2	0.008
HR control			
P_1_, beats/min	32.0 ± 4.4	34.5 ± 4.0	0.945
P_2_, mmHg^−1^	0.102 ± 0.019	0.074 ± 0.006	0.195
P_3_, mmHg	139.1 ± 4.6	136.1 ± 3.3	0.461
P_4_, beats/min	360.9 ± 8.4	361.0 ± 8.3	0.641
Neural arc			
P_1_, %	82.2 ± 9.0	73.4 ± 10.7	0.383
P_2_, mmHg^−1^	0.108 ± 0.014	0.107 ± 0.013	> 0.999
P_3_, mmHg	129.6 ± 4.4	125.6 ± 5.0	0.641
P_4_, %	20.2 ± 8.2	25.1 ± 10.1	0.078
Peripheral arc			
Intercept, mmHg	52.9 ± 7.9	38.8 ± 7.1	0.016
Slope, mmHg/%	0.678 ± 0.095	0.534 ± 0.088	0.016
SNA–SVR			
Intercept, mmHg·min/mL	1.235 ± 0.187	0.851 ± 0.123	0.008
Slope, mmHg·min·mL^−1^·%^−1^	0.010 ± 0.001	0.008 ± 0.001	0.109
SNA–AoF			
Intercept, mL/min	44.0 ± 3.0	44.9 ± 2.6	0.742
Slope, mL·min^−1^·%^−1^	0.080 ± 0.013	0.089 ± 0.027	0.742
Operating point			
AP, mmHg	112.5 ± 2.5	88.6 ± 2.9	0.008
SNA, %	89.4 ± 2.6	95.0 ± 6.3	0.148

Data are means ± SE (n = 8 rats). P_1_, response range; P_2_, slope coefficient; P_3_, midpoint pressure; P_4_, lower limit; SNA, sympathetic nerve activity; SVR, systemic vascular resistance; AoF, aortic flow; AP, arterial pressure. The P-values were derived from the Wilcoxon signed-rank test.

## Discussion

### Effects of vericiguat and SNP on the peripheral arc and SVR

Both vericiguat and SNP narrowed the range of the AP response and decreased the lower limit of the baroreflex total arc (Figs 3A and 5A). For both drugs, the AP reduction was primarily due to the downward change of the peripheral arc (Figs 3D and 5D). The change in the AP may be decomposed into changes in the SVR and AoF. Vericiguat halved the slope of the SNA–SVR relationship (Fig 3E), whereas SNP caused a near parallel downward shift in the SNA–SVR relationship (Fig 5E). Although both drugs act on the NO-sGC-cGMP pathway, the differential effects on the SNA–SVR relationship may be explained by differences in their modes of action. As mentioned in the Introduction, vericiguat acts independently of endogenous NO but also enhances sGC sensitivity to endogenous NO [7, 8, 25]. The endogenous NO production in the vascular endothelium increases at high AP levels because of the increased shear stress [26, 27]. Accordingly, the vasodilative effect of vericiguat might have become greater toward higher levels of AP and SNA (Fig 3E). It may also be possible that SNA augmented the NO-independent direct effect of vericiguat on the sGC-cGMP pathway. By contrast, SNP might have caused vasodilation irrespective of the levels of endogenous NO (Fig 3E). In an *in vitro* study, SNP showed a parallel rightward shift of the dose-response curve of the rat aorta to noradrenaline [28]. Although the lack of shear stress is a confounding factor in interpreting the results of the organ bath experiment, the parallel shift of the dose-response curve may be in line with the downward shift of the SNA–SVR relationship observed in the present study.

Vericiguat significantly increased the intercept and slope of the SNA–AoF relationship (Fig 3F). An afterload reduction by vasodilation might have contributed to the increased AoF because vericiguat has no significant effect on the ventricular contractility in rat heart Langendorff preparations [7]. By contrast, SNP hardly affected AoF (Fig 3F) despite the afterload reduction by vasodilation. Veins show greater sensitivity than arteries for SNP and nitroglycerin in the organ bath, though SNP exerts a more balanced effect on resistance and capacitance vessels *in vivo* [29]. In a canine right-heart bypass preparation [30], SNP decreased cardiac output under normal conditions because of the downward shift of the venous return curve. In the same study, induction of left ventricular failure by coronary occlusion caused a downward shift of the venous return curve. During the coronary occlusion, SNP did not significantly affect the venous return curve and increased cardiac output via afterload reduction. Hence, the balance between afterload reduction and preload reduction under a given set of conditions is a determinant of the effect of vasodilators on cardiac output. Vericiguat marginally increased the averaged CVP, whereas SNP did not. However, because we did not measure left ventricular function, we were unable to identify the exact mechanism for the differential effects of vericiguat and SNP on AoF.

There is limited clinical data demonstrating the impact of vericiguat on hemodynamics. In the VICTORIA trial [11], which showed the clinical benefits of vericiguat in HFrEF (heart failure with reduced ejection fraction) patients, the BP reduction in the vericiguat group was very small, which is considered one of the advantages of vericiguat over other guideline-directed medical therapies. The fact that vericiguat does not directly reduce SNA and increase cardiac output by decreasing LV afterload may help maintain BP. Meanwhile, in this study, vericiguat reduced BP by substantially decreasing SVR. The dose of vericiguat used in this experiment differed from the clinical dose and may have resulted in a greater reduction in SVR than in clinical situations. The daily dose of vericiguat is a maximum of 10 mg, which corresponds to 0.139 μg·kg^−1^·min^−1^ if we assume a body weight of 50 kg. Hence, the dose of 10 μg·kg^−1^·min^−1^ in the present study needs to be interpreted as a dose above the therapeutic range. In the SOCRATES-REDUCED trial [9], patients in the 10-mg vericiguat group experienced an increase in left ventricular ejection fraction. However, to our knowledge, there is no available data regarding the effect of vericiguat on cardiac output. As discussed above, the effect of a test drug could be determined by the balance between its vasodilative effects on arteries and veins.

### Effects of vericiguat and SNP on the neural arc and HR response

Although sGC exists ubiquitously in the whole body including the central nervous system [31], neither vericiguat nor SNP acutely affected the neural arc. SNP has been used with phenylephrine to perturb the AP and estimate the baroreflex-mediated SNA and HR responses [32, 33]. The presumption for this method is that SNP does not directly modulate the central processing of the arterial baroreflex. The fact that there is no significant effect of SNP on the baroreflex neural arc (Fig 5C) ascertains that SNP is appropriate for use as a test drug for estimating baroreflex function.

Vericiguat has little ability to cross the blood-brain barrier (BBB) [34]; and thus, the finding of no significant effect of intravenous vericiguat on the neural arc (Fig 3C) might have been predicted. Nevertheless, low penetration across the BBB is not necessarily the same as absence of central effects of a given agent. As an example, plasma angiotensin II does not cross the BBB under normal conditions but exerts a central effect by acting on brain areas that reside outside the BBB, such as the subfornical organ and the area postrema [35]. High levels of circulating angiotensin II caused a near parallel upward shift in the baroreflex neural arc [36].

In the present experimental settings, the HR response to the CSP change was mediated by the sympathetic nervous system because of vagotomy. Hence, no significant effects of vericiguat and SNP on the CSP–HR relationship (Figs 3B and 5B) are consistent with the finding of no significant effects of these drugs on the baroreflex neural arc. Further, the results on the HR response suggest that neither vericiguat nor SNP has a significant direct effect on the sinoatrial node. Rat heart Langendorff preparations also indicate no significant effect of vericiguat on HR [7].

### Limitations

Several limitations need to be mentioned. First, the experiment was performed on anesthetized rats, and careful interpretation is required when extrapolating the present results to conscious conditions and other species. Second, the drug effects were examined in normal rats. Because the level of endogenous NO may be decreased in diseased conditions, the effects of vericiguat and SNP on the peripheral arc in diseased conditions could be different from the present results. Studies using a rat model of myocardial infarction or Dahl salt-sensitive rats may be required to investigate the effects of vericiguat and SNP in chronic hyperadrenergic conditions. Third, only a single dose was tested in each protocol. There is room for argument that the difference in the SNA–SVR relationship between vericiguat and SNP could depend on the dose, and further studies focusing on the dose-response are warranted. Fourth, the intercept of SNA–SVR relationship under baseline was lower in the vericiguat protocol than in the SNP protocol for unknown reasons, which might have also affected the magnitude of the vasodilative effect. Finally, the first-in-class sGC stimulator riociguat was introduced as a novel treatment option for pulmonary hypertension [7]. We cannot find available literature regarding the effect of vericiguat on the vasodilative response of pulmonary arteries. Further studies are required to quantify the effect of vericiguat on the pulmonary vasculature.

## Conclusions

The effects of vericiguat on the open-loop baroreflex static characteristics were estimated and compared with those of SNP. Neither vericiguat nor SNP significantly affected the neural arc, suggesting the limited effects of these drugs on the central regulation of the sympathetic nervous system. Both vericiguat and SNP decreased the AP mainly through their peripheral effects. The vasodilative effect of vericiguat became greater toward high levels of SNA and AP, possibly reflecting the increased sGC sensitivity to endogenous NO. By contrast, the effect of SNP was more uniform over the range of SNA. The systematic analysis using the baroreflex open-loop procedure would help understand cardiovascular effects of vericiguat.

## Supporting information

S1 Data (XLSX)

S2 Data (XLSX)

S3 Data (XLSX)

S4 Data (XLSX)
